# Evolution of a Novel Appendage Ground Plan in Water Striders Is Driven by Changes in the *Hox* Gene *Ultrabithorax*

**DOI:** 10.1371/journal.pgen.1000583

**Published:** 2009-07-31

**Authors:** Abderrahman Khila, Ehab Abouheif, Locke Rowe

**Affiliations:** 1 Department of Biology, McGill University, Montreal, Quebec, Canada; 2 Department of Ecology and Evolutionary Biology, University of Toronto, Toronto, Ontario, Canada; Princeton University, Howard Hughes Medical Institute, United States of America

## Abstract

Water striders, a group of semi-aquatic bugs adapted to life on the water surface, have evolved mid-legs (L2) that are long relative to their hind-legs (L3). This novel appendage ground plan is a derived feature among insects, where L2 function as oars and L3 as rudders. The *Hox* gene *Ultrabithorax* (*Ubx*) is known to increase appendage size in a variety of insects. Using gene expression and RNAi analysis, we discovered that Ubx is expressed in both L2 and L3, but *Ubx* functions to elongate L2 and to shorten L3 in the water strider *Gerris buenoi*. Therefore, within hemimetabolous insects, *Ubx* has evolved a new expression domain but maintained its ancestral elongating function in L2, whereas *Ubx* has maintained its ancestral expression domain but evolved a new shortening function in L3. These changes in *Ubx* expression and function may have been a key event in the evolution of the distinct appendage ground plan in water striders.

## Author Summary

Water striders are derived semi-aquatic bugs that possess a remarkable diversity of leg lengths and shapes among species and between sexes, and the selective forces shaping this diversity are well studied. The transition to living on the water surface was accompanied by dramatic changes in the size and function of their legs. The mid-legs are disproportionately long and function as oars, whereas the hind-legs are shorter and function as rudders. We present evidence demonstrating that changes in the pattern of expression and function of the *Hox* gene *Ultrabithorax* are responsible for establishing the relative size differences between mid- and hind-legs in the water strider *Gerris buenoi*. These changes in *Ubx* expression and function may have been a key event in the evolution of the distinct appendage ground plan in water striders.

## Introduction

**Figure 1:**
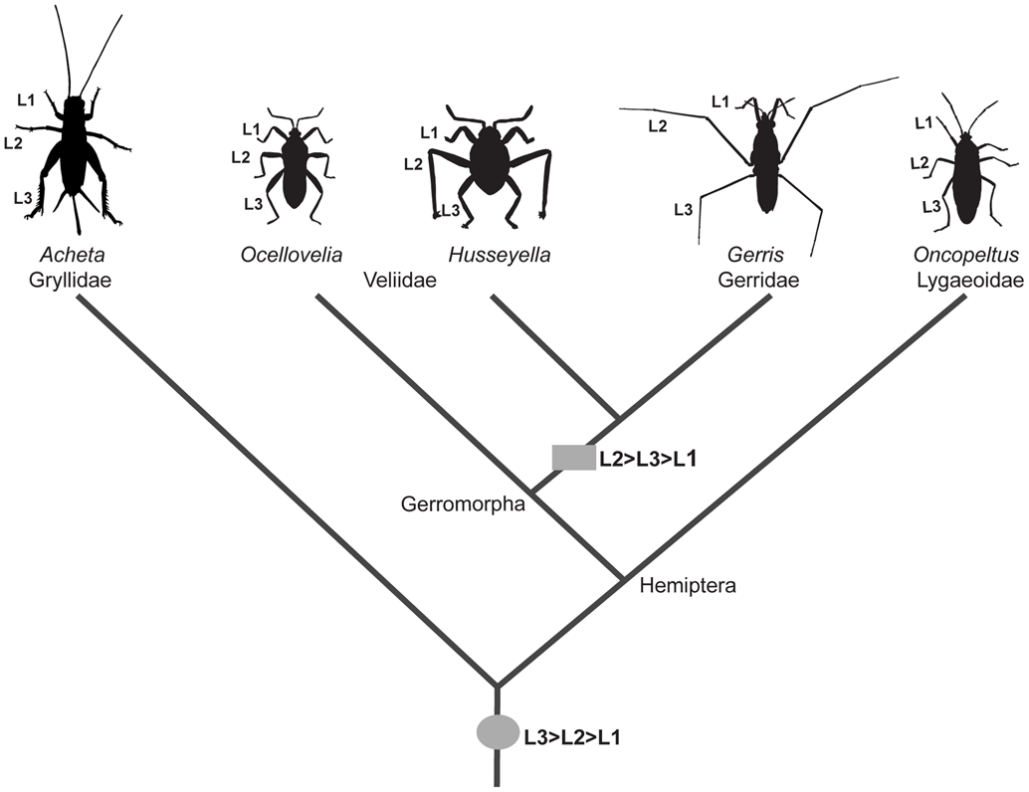
Ground plan of appendage morphology in insects. The common ancestor of Hemiptera most likely presents a universal insect ground plan where L3 is longer than L2, which is in turn longer than L1. The Lygaeoidae, which are terrestrial bugs such as *Oncopeltus*, represent the ancestral ground plan. Most semi-aquatic bugs (Gerromorpha) including the Veliidae, such as *Ocellovelia*, share a similar ground plan, which is associated with a mode of locomotion on water by alternating leg movements, similar to the terrestrial mode of locomotion. The Gerridae, such as *Gerris*, and some Veliidae, such as *Husseyella*, have evolved a derived ground plan where L2 is longer than L3, which is in turn longer than L1. This ground plan is an adaptation to a derived mode of locomotion on the water surface by means of oars (L2) and rudders (L3).

The diverse appendage morphologies found in insects constitute an important model for studying the developmental genetic mechanisms underlying morphological novelties [1]–[3]. Water striders are derived semi-aquatic bugs (Hemiptera, Gerromorphae, Gerridae), which possess a remarkable diversity of leg lengths and shapes among species and between sexes. We have a good understanding of the evolutionary forces that shape this diversity, including both adaptation to locomotion on the water surface [4],[5], and adaptations associated with mating [6],[7]. The combination of a striking diversity and an understanding of the forces shaping this diversity suggest that water striders provide an important context for understanding the developmental genetic basis of appendage diversification. Yet, there have been no developmental genetic studies of this group. Here we investigate the mechanisms underlying the distinctive appendage size ground plan in water striders. In most insects, the hind-legs (L3) are longer than the mid-legs (L2) and forelegs (L1), representing an L3>L2>L1 appendage size ground plan (Figure 1). Water striders have evolved a novel appendage plan where L2 are longer than L3 (L2>L3>L1; Figure 1). This ground plan has most likely evolved as a consequence of adapting to locomotion on the water surface [4]. L2 are disproportionately elongated and function as oars for propulsion, while L3 are shorter and function as rudders [7],[8]. L1 are the shortest among the three pairs, functioning primarily in prey handling.

In insects, including other hemipterans (e.g., *Oncopeltus fasciatus*), appendages differentiate from limb buds that are specified and elongated during embryonic development [2]. The final phase of appendage development consists of refining the allometric properties of each pair according to its segmental position and biological function [9]. The Hox gene *Ultrabithorax* (*Ubx*) is known to play multiple roles in defining specific morphological differences among the segments along the anteriorposterior body axis in arthropods, including appendage size, shape, and function [10]–[18]. In several hemimetabolous insect species, the spatial and temporal expression of *Ubx* correlates with the relative enlargement of the hind legs L3 [19]–[21]. *Ubx* expression in L3 segments causes their differential growth compared to those of L1 and L2, and the earlier *Ubx* is expressed in these segments, the more enlarged they become [21]. Furthermore, *Ubx* in *Drosophila* is expressed in both L2 and L3 during larval and pupal development, where it is required for establishing different patterns of trichome features within the femur [15],[22]. *Ubx* is also required for elongating the size of these legs as the loss of Ubx function causes a significant decrease in the size of L3 and a subtle decrease in the size of L2 [16]. We therefore tested whether *Ubx* plays a role in regulating the relative sizes of L2 and L3 legs in *G. buenoi*, and thus in the evolution of this derived ground plan within the Gerridae.

## Results/Discussion

### Differential leg sizes are established during embryonic development in *Gerris buenoi*

**Figure 2:**
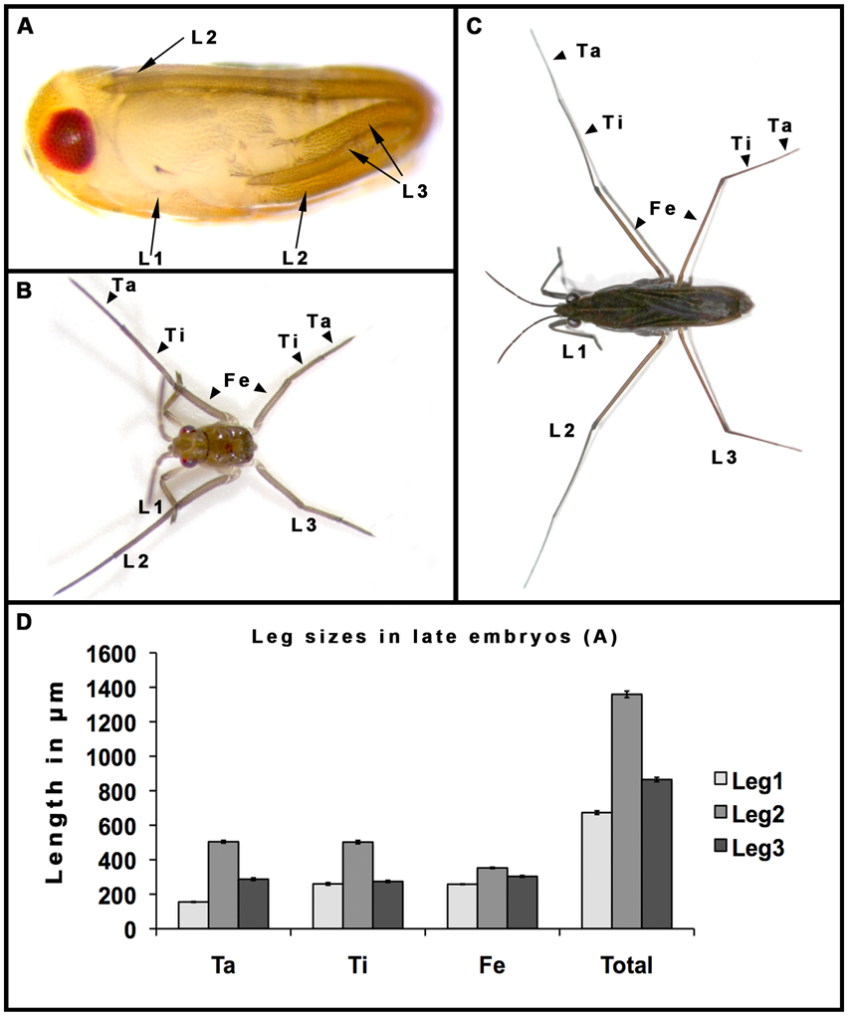
Leg arrangement, morphology, and size during various stages of *G. buenoi* ontogenesis. (A) Late embryo prior to hatching. L1 legs extend ventrally across the abdomen, to reach the junction between the femur and the tibia of L2. Both L2 legs extend in parallel in a ventral-to-dorsal arrangement and the tips of their tarsi reach the head of the embryo (L2 arrow). L3 legs, however, cross each other to extend in a lateral-to-lateral arrangement and the tips of their tarsi are tucked laterally between the proximal bases of L2 and L3 (L3 arrows). (B) First instar larva showing the differential sizes of the segments in each leg. Note the dramatic size elongation in L2 compared to L1 and L3. (C) *G. buenoi* adult female showing similar differences in the overall sizes of the legs compared to the larva. (D) Morphometric measurements of appendage sizes in the late embryonic stage, captured in (A). Data are expressed as means±SD. Note that the Tarsus (Ta), Tibia (Ti), and Femur (Fe) in L2 an L3 are the segments that are the most elongated. The average size variation is significantly different between the three appendages (F_2,27_ = 5866.264, P<0.05). The dynamics of growth of leg segments changes throughout ontogenesis (not shown). The tarsus and tibia of L2 are not significantly different in size (F_1, 18_ = 0.367, P = 0.552) and are both significantly longer than the femur (F_1, 18_ = 2526.094, P<0.01). In the adult, the femur of L2 is significantly longer than the tibia (F_1, 18_ = 15.533, P<0.025; Figure 2D), which is in turn significantly longer than the tarsus (F_1, 18_ = 50.522, P<0.001; Figure 2D). In L3 however, the tarsus is significantly longer than the tibia and significantly shorter than the femur throughout *G. buenoi* ontogenesis (F_2, 27_ = 55.308, P<0.01).

Size differences between the three pairs of legs are established during embryogenesis and can be visualized in late embryos (Figure 2A). At this stage, L2 is over one and a half times longer than L3 (Figure 2D). L2 and L3, due to their excessive length, extend in a stereotypic pattern along the body axes of late embryos (Figure 2A). L2 extend from the ventral towards the dorsal side (ventral-to-dorsal arrangement), whereas L3 extend from one lateral to the opposing lateral side of the embryo (lateral-to-lateral arrangement; arrows in Figure 2A). In first instar larvae (Figure 2B), the difference in size between L2 and L3 legs is comparable to the difference found between these two appendages in adults (Figure 2C).

**Figure 3:**
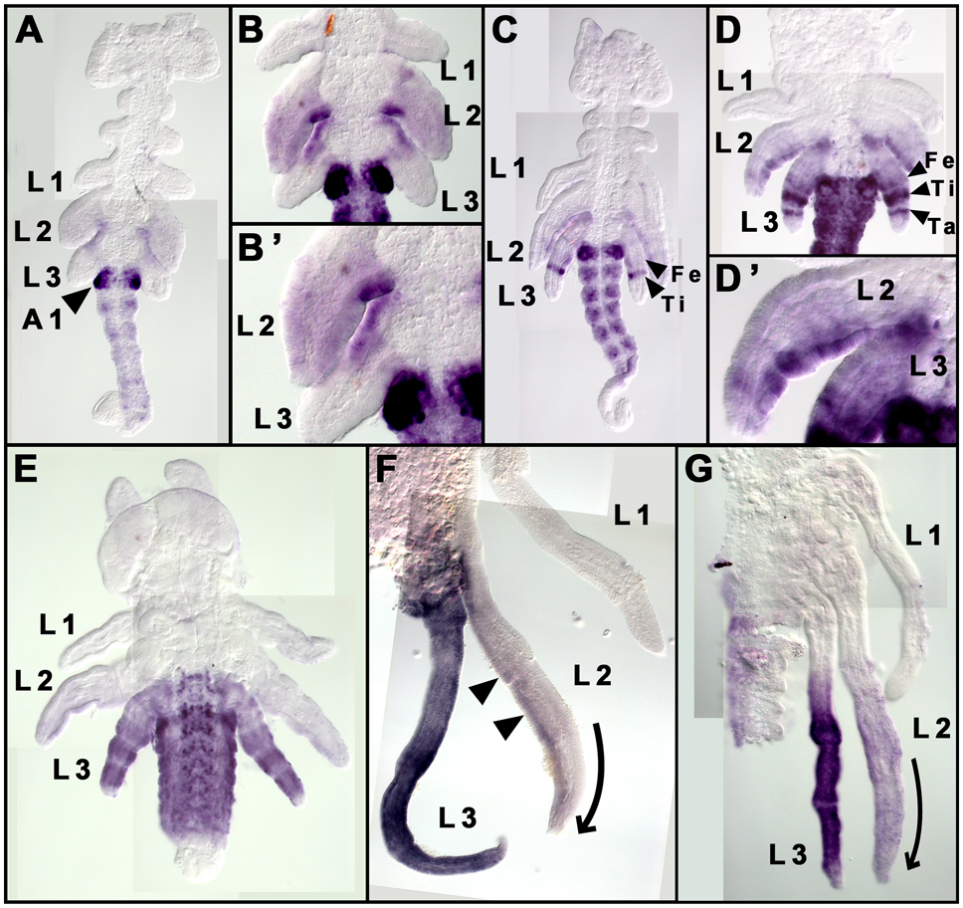
Expression patterns of Ubx and Abdominal-A (UbdA) proteins during *G. buenoi* embryogenesis. (A) Early segmented embryo where a faint UbdA is first seen in the legs (arrowhead indicates the strong UbdA accumulation in abdominal segment A1; anterior to the top). (B–B′) UbdA accumulates uniformly throughout the entire limb bud of L2, but is absent from the distal parts of L3 limb buds. (C–G) Dynamics of UbdA accumulation in both L2 and L3 legs (arrowheads indicate UbdA accumulation in the segments of each leg). (C) UbdA expression now appears as a strong stripe in the tibia and a faint stripe in the femur of L3. (D) This expression is followed by another strong stripe that corresponds to the Tarsus in L3. (D′) In L2, the levels of UbdA accumulation are stronger in the posterior relative to the anterior compartment. (E–G) This pattern continues in later embryonic stages where UbdA becomes strong and uniform in the whole L3 legs (E,F), and also persists in distinct levels between the anterior and posterior compartments in L2 (arrowheads in F). In the trunk, UbdA is first excluded from the thoracic segments (A–C), then appears later in faint levels in both T3 and the posterior compartment of T2 (D–E). Note the dynamics of L2 and L3 size development in (E–G), where L3 first reaches a longer size F before the size of L2 catches up (G). This dynamic of leg growth is consistent with the typical arrangement of these two legs along the embryo axis. Ti: Tibia, Ta: tarsus and Fe: Femur. Curved arrows in (F) and (G) indicate the differential growth between the anterior and the posterior compartments of L2, which results in the curving of this leg.

Insect appendages are generally subdivided into five segments, from proximal to distal: coxa, trochanter, femur, tibia, and tarsus. Elongation of the three distal leg segments, the femur, tibia, and tarsus, starts during *G. buenoi* embryogenesis and continues throughout subsequent developmental stages (Figure 2). In late embryos, the tibia and the tarsus of L2 are not significantly different in size, but both are significantly longer than the femur (Figure 2D). In contrast, the tibia in L3 is slightly but significantly shorter than the tarsus, and both tibia and tarsus are significantly shorter than the femur (Figure 3D). Together, these results show that the novel appendage size ground plan L2>L3>L1 is established early during *G. buenoi* development, and has evolved mostly through modifications of the three distal segments in L2 an L3.

### Ubx is expressed in both L2 and L3 in differential and dynamic patterns

The expression pattern of *Ubx* mRNA or both Ubx and Abdominal-A proteins (UbdA) in trunk segments of *G. buenoi* is conserved relative to other insects [17], [23]–[25]. The domain of Ubx expression expands anteriorly from abdominal segment A1 to the posterior of the second thoracic segment T2, whereas Abd-A is restricted to abdominal segments A2–A8. UbdA staining first appears in the abdominal segments only, strongly in A1 and weakly in A2 through A8 of early embryos (Figure 3A and 3B; Figure S1). Later in development, we observed a faint UbdA staining in the third thoracic segment T3 (Figure 3C and 3D), which expands in older embryos to the posterior compartment of the second thoracic segment T2 (Figure 3E), where it overlaps with the domain of expression of the segment polarity gene *engrailed* (data not shown).

In addition to the ancestral expression of Ubx in the hind legs L3 of hemimetabolous insects [19],[21],[25], we discovered a novel expansion of Ubx expression to L2 legs. The antibody detects Ubx in both L2 and L3 throughout embryonic development (Figure 3). This result suggests that *Ubx* may play a role in regulating the relative sizes of both appendages, and thus, may be responsible for the development of the derived appendage ground plan of water striders. Previous studies have shown a correlation between the spatial/temporal expression of *Ubx* and the enlargement of L3 in other insects [16],[20],[21]. We therefore expected a similar correlation between *Ubx* expression and the relative sizes of both L2 and L3, as well as the size of segments within these two legs in *G. buenoi*. However, we found Ubx spatial/temporal expression pattern to be consistent with some but not all aspects of leg size development within and between L2 and L3. Furthermore, we found that the levels of Ubx expression between L2 and L3 are not consistent with the relative sizes of these two legs. Between L2 and L3, Ubx expression is first detected uniformly throughout L2 but not in the distal part of L3 limb buds (Figure 3A, Figure 3B1 and 3B2), which is consistent with the larger size of L2 relative to L3. This suggests that the earlier timing of Ubx accumulation contributes to elongating L2 size relative to L3. However when Ubx appears in L3, its levels are greater than in L2 throughout the remainder of development (Figure 3C–3G), even though L2 is one and a half times longer than L3 (Figure 2D).

Within L2, the spatial/temporal expression of Ubx is not consistent with the size differences among the three distal segments. Ubx expression appears simultaneously and uniformly in all these three distal segments (Figure 3B and 3B′), even though the tarsus and the tibia are both longer than the femur (Figure 2D). Interestingly, L2 legs curve slightly in the embryo (curved arrow in Figure 3F and 3G) most likely through faster growth in the anterior compared to the posterior compartment. This differential growth in L2 compartments is likely to direct this leg to wrap around the embryo in a ventral-to-dorsal arrangement (Figure 2A). We uncovered higher levels of Ubx accumulation in the posterior relative to the anterior compartment of L2 legs throughout most of embryo development (Figure 3C–3F). Therefore, *Ubx* may also be involved either early in the differential growth of the two L2 compartments to direct the curving, or later in correcting growth differences between the two compartments so that L2 legs become straight at the end of embryo development.

Within L3, Ubx first accumulates in a strong stripe corresponding to the future tibia, and a faint stripe corresponding to the future femur (Figure 3C). It is only shortly after this developmental stage that a stripe corresponding the future tarsus appears (Figure 3D). This timing of Ubx expression in L3 is not consistent with the size differences between these segments, as the tibia is the shortest in this leg compared to both tarsus and femur. The three stripes of Ubx expression in L3 expand and become uniform in the whole leg including the two proximal segments, coxa and trochanter (Figure 3E and 3F), even though these two proximal segments are not elongated. Furthermore, the levels of Ubx accumulation within L3 are higher in the tibia and in the tarsus, but lower in the femur (Figure 3C and 3D), despite the tarsus and tibia being significantly smaller than the femur in both late embryos and adults (Figure 2D). Altogether, these results suggest that *Ubx* may specify the development of *G. buenoi* appendage ground plan by playing multiple and distinct roles in regulating the size differences between L2 and L3, and those of the segments within each of these legs.

### *Ubx* functions to elongate L2 and to shorten L3 in *G. buenoi*

To determine the function of *Ubx* in the water strider appendage ground plan, we used parental RNA interference. We injected adult females with *Ubx* double stranded RNA (ds-*Ubx*) to knockdown *Ubx* expression, and separately injected females with *yellow fluorescent protein* double stranded RNA (ds-*YFP*) as a negative control (Text S1 and Figure S1). Surprisingly, *Ubx* knockdown resulted in shorter L2 but longer L3 compared to their control counterparts.

**Figure 4:**
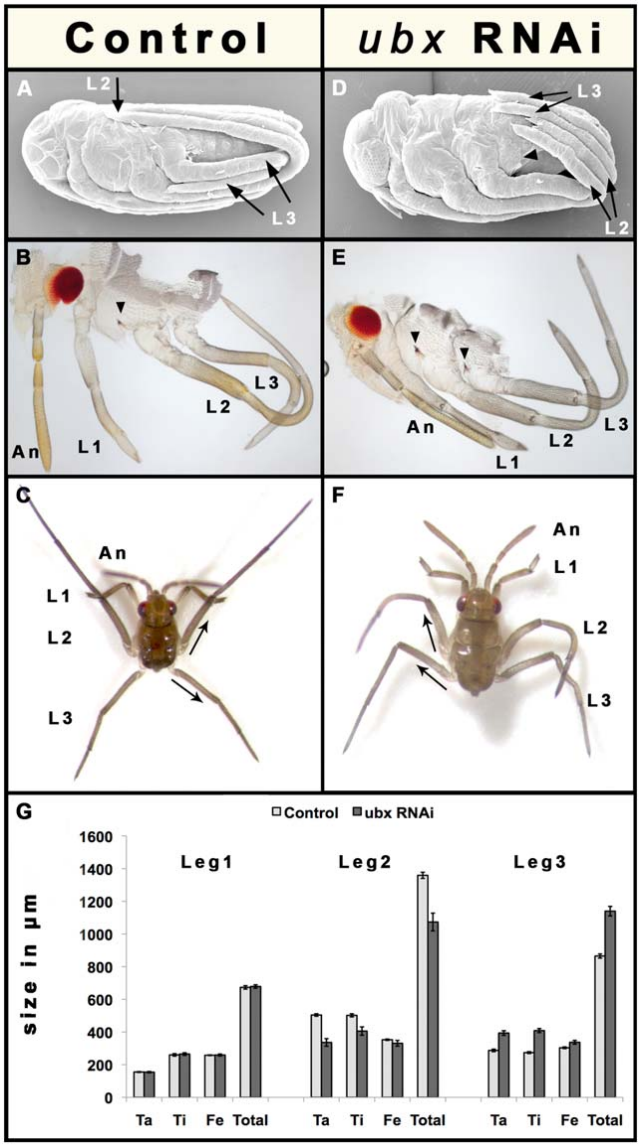
*Ubx* RNAi phenotypes in *G. buenoi*. (A,B) *YFP* control embryos and C *YFP* control first instar larvae show wild type development. (D,E) *Ubx* RNAi embryos and (F) *Ubx* RNAi first instar larvae show a variety of phenotypes affecting segment identity and leg sizes. In control embryos, L2 legs are longer and adopt a ventral-to-dorsal arrangement, whereas L3 are shorter and adopt a lateral-to-lateral arrangement (A). In ds-*Ubx* embryos, L2 legs look now shorter than L3 and both pairs adopt a ventral-to-lateral arrangement (B). The effect of *Ubx* depletion on the sizes of L2 and L3 is more obvious in dissected embryos (B,E), where L2 becomes shorter and L3 longer in *Ubx* embryos E compared to the controls (B). Note the appearance of the spiracle that characterizes L2 in *Ubx*-depleted L3 (arrowheads in B and E). *Ubx* RNAi larvae in (F) bear L2 and L3 legs that are similar in size and morphology compared to control larvae (C). Note that in *Ubx* RNAi larvae (E), L2 are curved towards the posterior and L3 femurs point towards the head, which is a usual posture for wild type L2 (arrows in F). (G) Comparison of the sizes of appendage segments between control and *Ubx* RNAi late embryos (in A and B). Data are expressed as means±SD. *Ubx* RNAi causes size shortening in L2 and size elongation in L3. The leg segments that are the most affected by *Ubx* depletion are the tarsi and the tibias in both appendages.

In L2, *Ubx* depletion results in a 20% shortening relative to the control embryos (F_1, 18_ = 205.326, P<0.001). The sizes of the tarsi, tibias, and femurs within L2 are significantly reduced (F_1,18_ = 78.093, P<0.001; Figure 4G), although the tarsi and the tibias are affected to a much greater extent than the femur. Furthermore, L2 legs in ds-*Ubx* first instar larvae are curved towards the posterior (Figure 4F). This result may be accounted for by the higher levels of Ubx accumulation we observed in the posterior relative to the anterior compartment of this leg (Figure 3C–3F). During embryonic development, as L2 legs are elongating they curve dorsally and wrap around the embryo in a stereotypic ventral-to-dorsal arrangement (Figure 2A). This dorsal curvature may occur through faster growth of the anterior relative to the posterior compartment as L2 elongates (curved arrow in Figure 3F and 3G). After L2 has curved towards the dorsal side of the embryo and before the nymph emerges, L2 must straighten while it continues to elongate. The processes of straightening and elongation of L2 may be affected by the stronger Ubx expression we observed in the posterior, causing this compartment to now grow faster relative to the anterior. Thus, in the absence of *Ubx*, the growth difference between the two L2 compartments is not corrected resulting in L2 that are curved instead of straight (Figure 4F), and that are shorter relative to the controls (Figure 4D). Therefore, *Ubx* is necessary for straightening the initial curvature as well as elongating the size of L2 legs.

In contrast to our results for L2, we observed a 22% elongation of L3 in ds-*Ubx* embryos compared to control embryos (F_1, 18_ = 865.620, P<0.001). Within L3, the tarsus and the tibia are the most elongated relative to the femur in ds-*Ubx* animals (Figure 4G), and these are the two segments with the higher levels of Ubx expression in wild type (Figure 3C and 3D). Therefore, *Ubx* functions to reduce the size of L3, primarily through reducing the sizes of the tibia and the tarsus (Figure 4G). This is opposite to the function of *Ubx* in L2.

Unlike in the second thoracic segment, where *Ubx* primarily elongates the size of L2, *Ubx* plays multiple and specific functions to establish the distinct identity of the third thoracic segment, including the reduction of L3 size (Figure 4; Text S1 and Figure S2). These functions include: (1) establishing morphological differences of the spiracles found on the base of L2 and L3, because both legs in ds-*Ubx* animals now exhibit a spiracle at their base that is characteristic to wild type L2 legs only (Figure 4E and Figure S2D); (2) directing L3 to wrap in a lateral-to-lateral arrangement, because in ds-*Ubx* embryos both L3 and L2 legs are now arranged ventral-to-dorsal as in wild type L2 but not L3 legs (Figure 4D and Figure S2C, S2D); and (3) orienting the L3 femurs to point posteriorly, because L3 femurs in live ds-*Ubx* first instar larvae now point anteriorly, which is the posture for L2 legs in wild type (compare arrow directions in Figure 4C and 4F).

### Conclusions

Our results show that *Ubx* establishes the appendage ground plan in water striders through elongating L2, and through multiple functions that establish the identity of the third thoracic segment, including the shortening of L3. In other insects that present the common L3>L2>L1 appendage ground plan such as *O. fasciatus* and *Acheta domesticus*, *Ubx* is expressed in L3 only and functions to elongate its size [20]. Therefore in the novel L2>L3>L1 ground plan of water striders, *Ubx* has evolved a new expression domain but maintained its ancestral elongating function in L2, whereas in L3, *Ubx* has maintained its ancestral expression domain but evolved a new shortening function.

The gain of *Ubx* expression in L2 may have evolved through the ability of *Ubx* to respond to one or several upstream regulators in this leg. One possibility to explain the evolution of this pattern may be that L2 has gained the expression of a factor(s) that can either promote the activation or alleviate the repression of *Ubx* in this leg [26],[27]. Another possibility may be that *Ubx* cis-regulatory elements have been modified such that these elements are now accessible for an activator or inaccessible for a repressor in L2 [27]–[30]. When these changes in *Ubx* expression began to randomly appear, they resulted in longer L2, which may have enhanced or promoted the specialized function of this pair as oars.

In addition to elongating L2, the evolution of the L2>L3>L1 appendage ground plan may have involved a shortening of L3 relative to L2. This is supported by our observation that in *G. buenoi* the function of *Ubx* is to shorten L3 legs. One possibility may be that Ubx in L3 has gained new downstream targets [18],[31], or may have gained the ability to associate with different co-factors [14],[32],[33], so that the outcome of these interactions is opposite to that in L2, resulting in the development of shorter legs. Another possibility could be that the differential timing and levels of Ubx expression [13],[21],[34] in L3 relative to L2 may be responsible for this opposing function of *Ubx* in regulating the size of these two legs. As in L2, this new *Ubx* function in shortening L3 may have enhanced or promoted the function of L3 as rudders.

Although most insects possess the common L3>L2>L1 plan, multiple independent transitions to L2>L3>L1 appear to have occurred in the Veliidae, a group that is basal to the Gerridae [4],[35] (Figure 1). These transitions may have involved repeated parallel changes in the expression and function of *Ubx* to elongate L2 and shorten L3. It is only in the derived Gerridae where the L2>L3>L1 plan becomes a general feature, which may suggest that this ground plan has been a key adaptation during the evolution of this group [4],[35]. Future studies on semi-aquatic bugs should focus on whether transitions to the derived appendage ground plan are generally associated with parallel changes in *Ubx* expression and function, as well as on elucidating the molecular mechanisms underlying the opposite effect of *Ubx* in L2 compared to L3.

## Materials and Methods

### Animals

*G. buenoi* females were collected from a pond in Toronto, Ontario, Canada. Water striders were reared in water tanks and fed with live *Drosophila*. Females lay eggs that are glued length-wise to floating pieces of *Styrofoam* by a gelatinous substance, which swells in water [36]. These eggs can be incubated at 25–27°C to allow embryo development.

### *Ubx* cloning

Total RNA was extracted from late embryos and early pro-larvae, and used as template in a first strand complementary DNA synthesis reaction (Invitrogen). This first strand cDNA pool was used as a degenerate PCR template to amplify a 500 base pairs (bp) *G. buenoi Ubx* fragment. The following forward and reverse *Ubx* degenerated primers were synthesized based on an *Ubx* sequence alignment from closely related insects: Forward: 5′- TAYGCCGCKGTKGTGGCAGCCGC-3′ from HQNGYAAV and reverse: 5′- TTCATKCGY CGGTTTTGGAACC -3′ from WFQNRRMK. *G. buenoi Ubx* sequence can be retrieved in Genbank under the accession number: FJ460166.

### Leg measurements and statistical analyses

All measurements were performed on a sample size of 10 animals (i.e., n = 10) using a Zeiss dissecting scope and Axiovision software. We first performed a model II single classification analysis of variance (ANOVA; [37]) to determine whether or not there is a statistically significant difference between the mean sizes of (1) L1, L2 and L3; and between (2) tarsus, tibia and femur of L1; tarsus, tibia and femur of L2, and tarsus, tibia and femur of L3; and finally between (3) the tarsi between all three legs; the tibias between all three legs; and the femurs between all three legs. We then performed a two-way mixed model ANOVA [37] to determine whether or not there is a statistically significant difference between the mean sizes of (1) L1, L2 and L3 in control and ds-*Ubx* treatment; between (2) tarsi of L1, L2 and L3 in control and ds-*Ubx* treatment; between (3) tibias of L1, L2 and L3 in control and ds-*Ubx* treatment, and finally between (4) femurs of L1, L2 and L3 in control and ds-*Ubx* treatment. All statistical analyses were performed using SPSS (SPSS Inc).

### Embryo dissection and fixation

*G. buenoi* embryos were treated with bleach to remove the chorion, then fixed in 4% formaldehyde in PTW (1× PBS; 0.1% tween-20) and heptane for 20 min. Embryos are then washed several times in freezer-cold methanol and stored in methanol at −20°C. Alternatively, embryos can be dissected out of the yolk then fixed for 20 min in 4% formaldehyde in PTW (1× PBS; 0.1% tween-20).

### *Ubx* in situ hybridization

*Ubx* in situ hybridization is performed using a DIG-labeled *Ubx* anti-sense RNA probe (Roche). Fixed embryos are rehydrated in decreasing methanol concentrations in PTW then washed several times in PBT (1× PBS; 0.3% Triton). The remnants of chorions are removed, manually using fine forceps, followed by a 2 min Proteinase K (50 µg/ml) treatment in PTW. Embryos are washed two times in PBT then incubated 2 min in 2 mg/ml Glycine in PBT to inactivate Proteinase K. Embryos are washed several times in PBT, post-fixed in 4% formaldehyde for 20 min, then washed several times again in PBT. Embryos are pre-hybridized one hour in hybridization solution (50% formamide, 5× SSC pH 6.5, 50 ug/ml salmon sperm DNA, 50 µg/ml Heparin and 0.1% Tween-20) at 58°C, then hybridized over-night with *Ubx* probe in 100 µl hybridization solution at 58°C. Embryos are washed in decreasing concentrations of hybridization solution in PBT at 58°C, then five times in PBT at room temperature followed by a one hour blocking step in PAT (1× PBS; 1% Triton-X 100 and 1% bovine serum albumin). Embryos are then incubated two hours at room temperature with the anti-DIG antibody, conjugated with Alkaline Phosphatase. *Ubx* expression is revealed using NBT/BCIP as substrates for the Alkaline Phosphatase.

### Ubx/Abdominal-A antibody staining

Ubx/Abdominal-A antibody staining is performed using the FP6.87 antibody that recognizes both Ubx and Abdominal-A proteins [19]. Fixed embryos are manually dissected to remove the remnants of the chorion, and then incubated in a blocking solution (1× PBS; 0.1% Triton-X 100; 0.1% BSA and 10% Normal Goat Serum) for one hour at room temperature. Blocked embryos are then incubated with the FP6.87 antibody at 1/5 dilution over-night at 4°C. Embryos are then washed five times in blocking solution then incubated with an anti-mouse secondary antibody conjugated to horse radish peroxydase for two hours at room temperature. Embryos are washed five times in PBT, then UbdA expression revealed in a reaction solution containing DAB, NiCl2 and 0.002% H2O2.

### *Ubx* parental RNAi

The synthesis of *Ubx* double stranded RNA (ds-*Ubx*) was performed as described in [38]. The following *Ubx* forward 5′-TAATACGACTCACTATAGGGAGACCACGTggcagccgcatgtaagctatatt - 3′ and reverse 5′-TAATACGACTCACTATAGGGAGACCACGTttggtacctcgtatatgtttgtc-3′ primers containing T7 promoter sequence (capital letters) were used to clone a fragment that is flanked by T7 promoter from each side. This fragment was used as a template for in vitro transcription using T7 RNA polymerase, generating both sense and anti-sense transcripts, at 37°C. Complementary single RNA strands are automatically annealed into double stranded RNA (dsRNA) while the reaction progresses without any further treatment. dsRNA is then purified using Qiagen RNeasy purification kit and eluted in Spradling injection buffer [39]. *G. buenoi* adult females were anesthetized using carbon dioxide, immobilized on double sticky tape and injected with 1.5–2 µl *Ubx* dsRNA or *YFP* dsRNA (as negative control) at ∼2 µg/µl concentration. Injected females were replaced on water tanks; embryos collected on floating Styrofoam and allowed to develop at room temperature. Embryos were screened for phenotypes morphologically by examining leg sizes and segment transformation.

## Supporting Information

Figure S1 Specificity and phenotype frequency of Ubx RNAi in *G. buenoi*. (A) Embryo from a female injected with YFP double-stranded RNA as a control, stained for Ubx/Abd-A proteins. At this stage, Ubx is expressed in the hind-legs and strongly in the boundary between T3 and A1 (arrowhead), whereas Abd-A is expressed in the abdominal segments A2–A7. (B) Embryo of a similar developmental stage from a female injected with Ubx double-stranded RNA, also stained for Ubx/Abd-A. Note that AbdA expression persists in the abdominal segments, while Ubx is no longer expressed neither in the boundary between A1 and T3 (arrowhead) nor in the hind-legs. This suggests that our Ubx dsRNA is highly specific and does not interfere with AbdA expression. (C) Ubx RNAi phenotype count based on homeotic defects observed in the trunk segments and their corresponding legs of late embryos and early emerged larvae. *G. buenoi* RNAi efficiency was higher than 90%, while no Ubx-specific phenotypes were found in the YFP control.
(1.41 MB TIF)

Figure S2 Effects of Ubx RNAi on the identity of *G. buenoi* thoracic and abdominal segments. (A) Dorsal view of a control embryo showing the length of the trunk segments T1 through A1. (B) Lateral view of the same embryo, showing spiracle that characterizes the base of L2 (arrowhead) as well as the ventral-to-dorsal stereotypic arrangement of L2 and the lateral-to-lateral arrangement of L3 appendages (arrows). Note that segment T3 also possesses a spiracle, which is smaller and distinguishable from that of L2. (C) Dorsal view of an Ubx-depleted embryo, showing an increase in the length of both T3 and A1 segments. (D) Lateral view of the same embryo, showing that both segments T3 and A1 now have developed the same T2-specific spiracle (arrowheads). Note in both (C) and (D) that L3 appendage now adopts a ventral-to-dorsal arrangement characteristic to L2. (E) Comparison of the lengths of trunk segments T2, T3 and A1 between control and Ubx RNAi embryos. Both segments T3 and A1 exhibit a dramatic increase in length in Ubx-depleted embryos suggesting that these segments now exhibit morphological features that resemble T2.
(1.80 MB TIF)

Text S1 Supplementary text.
(0.07 MB DOC)
