# Supplementary figures and images for: Evolution of a Novel Appendage Ground Plan in Water Striders Is Driven by Changes in the Hox Gene Ultrabithorax
Source: PLoS Genet. 2009 Jul 31;5(7):e1000583. doi: 10.1371/journal.pgen.1000583 (PMC2709915; doi:10.1371/journal.pgen.1000583)

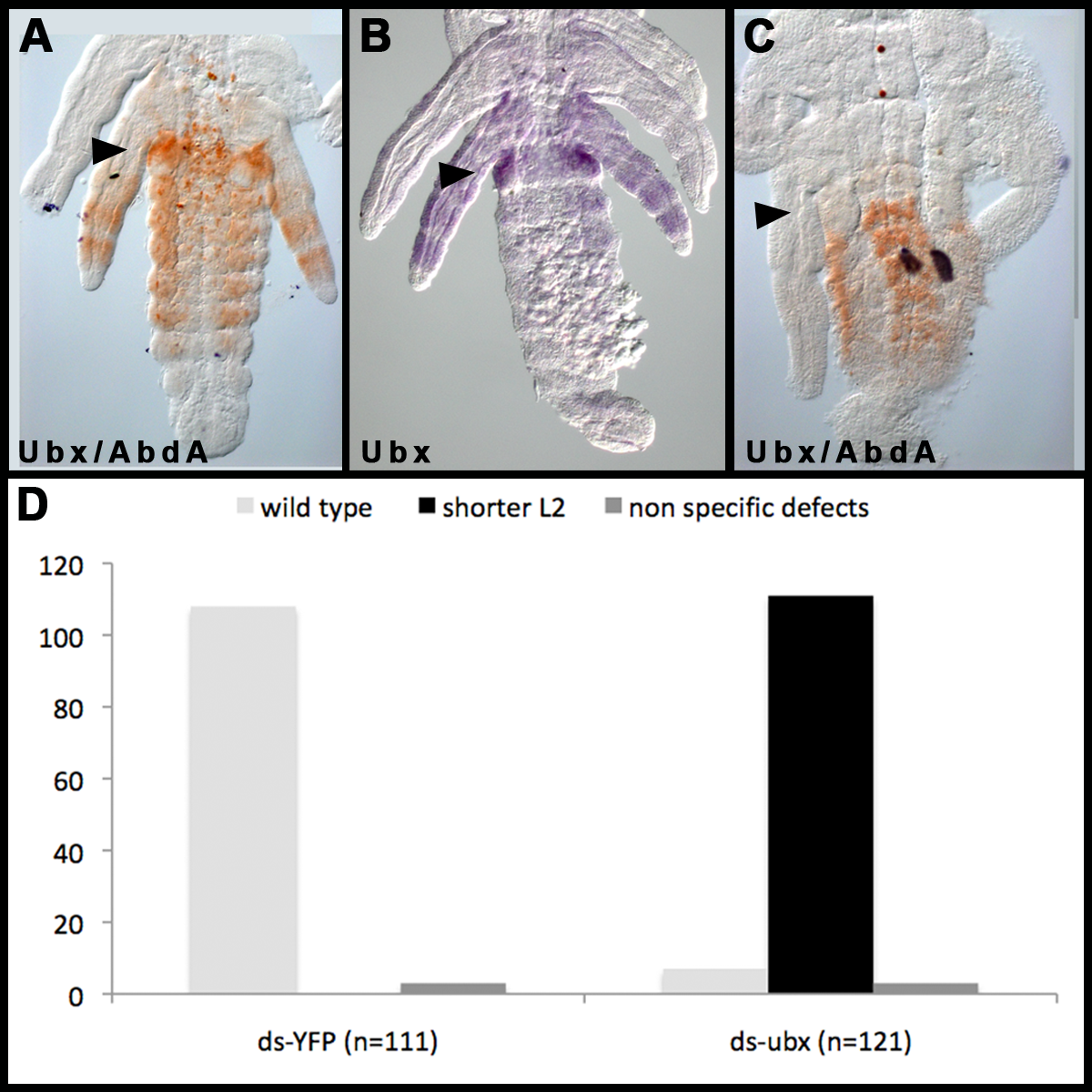

Supplement: Figure S1 — Specificity and phenotype frequency of Ubx RNAi in G. buenoi. (A) Embryo from a female injected with YFP double-stranded RNA as a control, stained for Ubx/Abd-A proteins. At this stage, Ubx is expressed in the hind-legs and strongly in the boundary between T3 and A1 (arrowhead), whereas Abd-A is expressed in the abdominal segments A2–A7. (B) Embryo of a similar developmental stage from a female injected with Ubx double-stranded RNA, also stained for Ubx/Abd-A. Note that AbdA expression persists in the abdominal segments, while Ubx is no longer expressed neither in the boundary between A1 and T3 (arrowhead) nor in the hind-legs. This suggests that our Ubx dsRNA is highly specific and does not interfere with AbdA expression. (C) Ubx RNAi phenotype count based on homeotic defects observed in the trunk segments and their corresponding legs of late embryos and early emerged larvae. G. buenoi RNAi efficiency was higher than 90%, while no Ubx-specific phenotypes were found in the YFP control. (1.41 MB TIF) [file pgen.1000583.s001.tif]

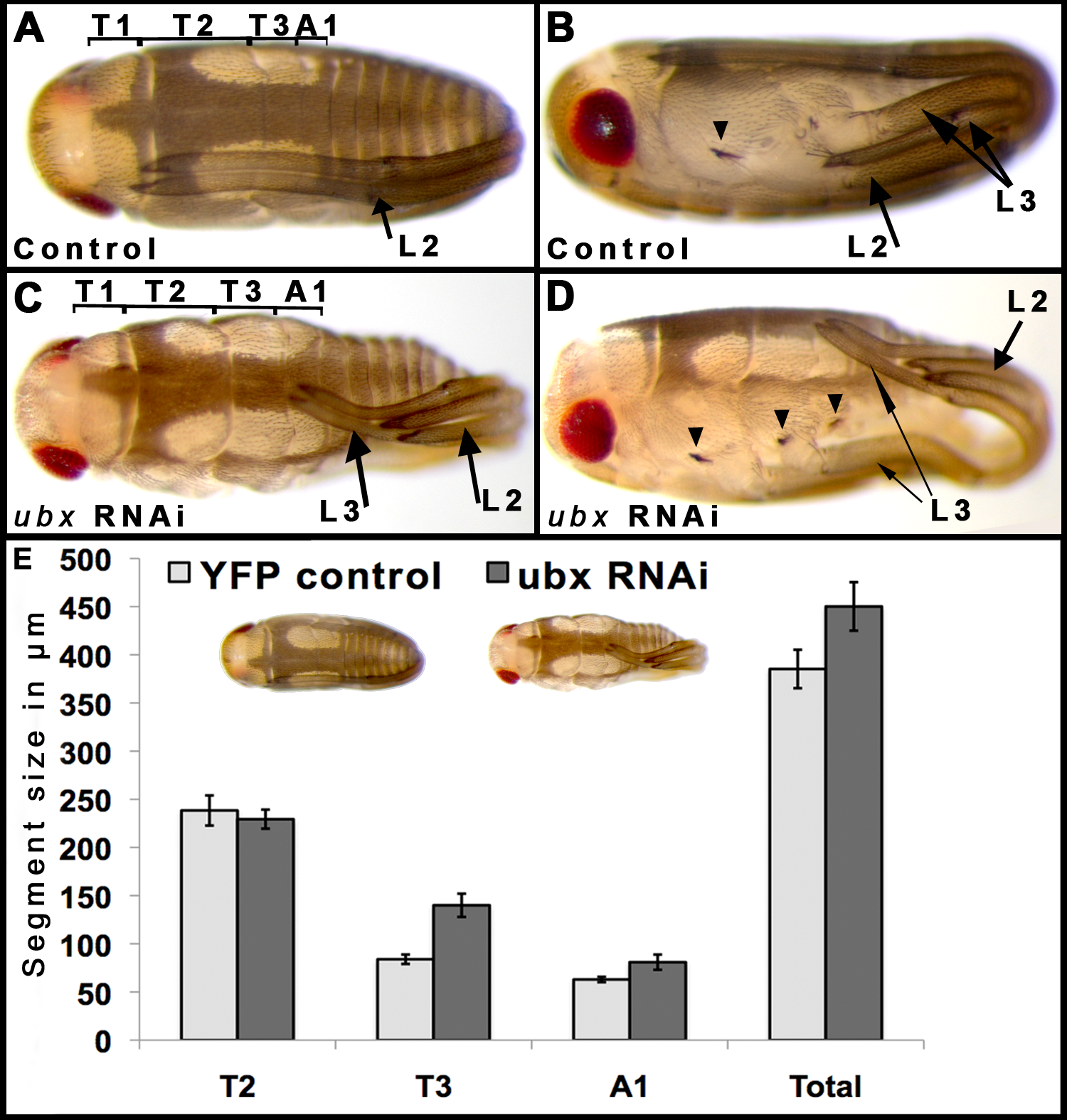

Supplement: Figure S2 — Effects of Ubx RNAi on the identity of G. buenoi thoracic and abdominal segments. (A) Dorsal view of a control embryo showing the length of the trunk segments T1 through A1. (B) Lateral view of the same embryo, showing spiracle that characterizes the base of L2 (arrowhead) as well as the ventral-to-dorsal stereotypic arrangement of L2 and the lateral-to-lateral arrangement of L3 appendages (arrows). Note that segment T3 also possesses a spiracle, which is smaller and distinguishable from that of L2. (C) Dorsal view of an Ubx-depleted embryo, showing an increase in the length of both T3 and A1 segments. (D) Lateral view of the same embryo, showing that both segments T3 and A1 now have developed the same T2-specific spiracle (arrowheads). Note in both (C) and (D) that L3 appendage now adopts a ventral-to-dorsal arrangement characteristic to L2. (E) Comparison of the lengths of trunk segments T2, T3 and A1 between control and Ubx RNAi embryos. Both segments T3 and A1 exhibit a dramatic increase in length in Ubx-depleted embryos suggesting that these segments now exhibit morphological features that resemble T2. (1.80 MB TIF) [file pgen.1000583.s002.tif]
