# Supplementary material for: Evolution of a Novel Appendage Ground Plan in Water Striders Is Driven by Changes in the Hox Gene Ultrabithorax
Source: PLoS Genet. 2009 Jul 31;5(7):e1000583. doi: 10.1371/journal.pgen.1000583 (PMC2709915; doi:10.1371/journal.pgen.1000583)
Supplement: Text S1 — Supplementary text. (0.07 MB DOC) [file pgen.1000583.s003.doc]

**Text S1**

***Ubx* RNAi specificity and frequency in *G. buenoi***

To analyze *Ubx* function in *G. buenoi* embryos, we isolated the gene *Ubx* and conducted a knockdown of its mRNA through parental RNAi [1-3]. Since this is the first RNAi analysis in this species, we wanted to confirm whether injection of *Ubx* double-stranded RNA (ds-*Ubx*) would result in specific *Ubx* depletion. We then stained ds-*Ubx* embryos and ds-YFP controls with the FP6.87 anti-Ubx/abdA antibody, which targets both Ubx and Abd-A proteins [4]. ds-YFP embryos showed wild type Ubx protein (Figure S1A) and mRNA (Figure S1B) expression in T3 including the hind legs, and strongly in A1, in addition to wild type Ubd-A protein accumulation in the abdominal segments A2-A8 (Figure S1A). However, in ds-*Ubx* embryos, Ubx protein is no longer expressed neither in the hind-legs nor in A1 segment, whereas Abd-A expression in the abdominal segments is not affected (Figure S1C). This suggests that our ds-RNA is highly specific to *Ubx* and does not interfere with the expression of other Hox genes. The effect of ds-*Ubx* is highly efficient through parental injection and yielded over 90% *Ubx* phenotypes, related to segment identity and leg size, whereas the majority of ds-YFP embryos showed a wild type development (Figure S1D). A small fraction of embryos in both ds-*Ubx* and ds-YFP exhibit variable unknown developmental defects.

***Ubx* depletion transforms the identity of both thoracic segment T3 and Abdominal segment A1 to the likeness of T2**

The second thoracic segment T2 in control embryos is significantly larger than the third thoracic segment T3 and the first abdominal segment A1 (F_1, 27_=1502.106, P<0.001; Figure S2 A and E). T2 also presents in the base of its corresponding legs L2 a characteristic spiracle that is easily distinguishable from that of segment T3 (arrowhead in Figure 4B and Figure S2B). A non-significant decrease in T2 size was observed between control and *Ubx*-depleted embryos (F_1, 18_=2.2227, P=0.153; compare T2 in Figure S2A, C and E), indicating a small requirement for *Ubx* in defining the morphology of that segment and that the function of *Ubx* in T2 is primarily to control the size of L2 appendage. We observed, however, a significant size increase in T3 (F_1, 18_=238.156, P<0.001; 67% increase) as well as A1 (F_1, 18_=52.397, P<0.001; 28 % increase) in *Ubx* knockdown compared to the controls (compare T3 and A1 in Figure S2A, C and E). We also observed the appearance of the spiracle that characterizes segment T2 of wild type embryos in both ds-*Ubx* T3 and A1 segments (arrowheads in Figure 4E and Figure S2D). This indicates that *Ubx* functions to specify the distinct morphology of both segments T3 and A1 to be different from that of T2, consistent with *Ubx* depletion in *O. fasciatus* [5]. Although most ds-*Ubx* larvae develop small ectopic limb buds (arrowhead in Figure 4D), we have not obtained any, however, with elongated appendages in the transformed A1 segment, suggesting that *Ubx* depletion is insufficient to de-block full leg development in *G. buenoi* abdominal segment A1. Altogether, these results suggest that the function of *Ubx* in defining the thoracic identity of segment T3 and the abdominal identity of segment A1 is a conserved feature across insects [5-8].

**References:**

1. Bucher G, Scholten J, Klingler M (2002) Parental RNAi in Tribolium (Coleoptera). Curr Biol 12: R85-86.

2. Hughes CL, Kaufman TC (2000) RNAi analysis of Deformed, proboscipedia and Sex combs reduced in the milkweed bug Oncopeltus fasciatus: novel roles for Hox genes in the hemipteran head. Development 127: 3683-3694.

3. Khila A, Grbic M (2007) Gene silencing in the spider mite Tetranychus urticae: dsRNA and siRNA parental silencing of the Distal-less gene. Dev Genes Evol 217: 241-251.

4. Kelsh R, Weinzierl RO, White RA, Akam M (1994) Homeotic gene expression in the locust Schistocerca: an antibody that detects conserved epitopes in Ultrabithorax and abdominal-A proteins. Dev Genet 15: 19-31.

5. Herke SW, Serio NV, Rogers BT (2005) Functional analyses of tiptop and antennapedia in the embryonic development of Oncopeltus fasciatus suggests an evolutionary pathway from ground state to insect legs. Development 132: 27-34.

6. Angelini DR, Liu PZ, Hughes CL, Kaufman TC (2005) Hox gene function and interaction in the milkweed bug Oncopeltus fasciatus (Hemiptera). Dev Biol 287: 440-455.

7. Carroll SB, Laymon RA, McCutcheon MA, Riley PD, Scott MP (1986) The localization and regulation of Antennapedia protein expression in Drosophila embryos. Cell 47: 113-122.

8. Mahfooz N, Turchyn N, Mihajlovic M, Hrycaj S, Popadic A (2007) Ubx regulates differential enlargement and diversification of insect hind legs. PLoS ONE 2: e866.
